# Estimation of Spatial-Temporal Distribution of Grazing Intensity Based on Sheep Trajectory Data

**DOI:** 10.3390/s22041469

**Published:** 2022-02-14

**Authors:** Xiantao Fan, Chuanzhong Xuan, Mengqin Zhang, Yanhua Ma, Yunqi Meng

**Affiliations:** 1 College of Mechanical and Electrical Engineering, Inner Mongolia Agricultural University, No. 306 Zhaowuda Road, Saihan District, Hohhot 010018, China; xtfan15053474683@163.com (X.F.); zhangmq@163.com (M.Z.); yanhuama@126.com (Y.M.); yunqimeng2019@163.com (Y.M.); 2 Inner Mongolia Engineering Research Center for Intelligent Facilities in Prataculture and Livestock Breeding, No. 306 Zhaowuda Road, Saihan District, Hohhot 010018, China

**Keywords:** trajectory data, grazing sheep, neural network, grazing intensity, spatial-temporal distribution

## Abstract

In the arid grasslands of northern China, unreasonable grazing methods can reduce the water content and species numbers of grassland vegetation. This project uses solar-powered GPS collars to obtain track data for sheep grazing. In order to eliminate the trajectory data of the rest area and the drinking area, the kernel density analysis method was used to cluster the trajectory point data. At the same time, the vegetation index of the experimental area, including elevation, slope and aspect data, was obtained through satellite remote sensing images. Therefore, using trajectory data and remote sensing image data to establish a neural network model of grazing intensity of sheep, the accuracy of the model could be high. The results showed that the best input parameters of the model were the combination of vegetation index, sheep weight, duration, moving distance and ambient temperature, where the coefficient of determination $R^{2}=0.97$, and the mean square error *MSE* = 0.73. The error of grazing intensity obtained by the model is the smallest, and the spatial-temporal distribution of grazing intensity can reflect the actual situation of grazing intensity in different locations. Monitoring the grazing behavior of sheep in real time and obtaining the spatial-temporal distribution of their grazing intensity can provide a basis for scientific grazing.

## 1. Introduction

Grassland resources are extremely rich with various natural grasslands of about 400 million hectares in China, which account for about 41% of the land area [1]. Inner Mongolia grassland belongs to the northern temperate zone, and the total area ranks first among the five grasslands in China. Due to the advantages of the natural conditions, Inner Mongolia has become an important livestock production base in China [2]. With the rapid development of animal husbandry, the situation of grassland degradation is severe, and grassland desertification caused by overgrazing leads to a serious decline in grassland productivity [3,4,5,6]. By monitoring the grazing behavior of sheep with modern technology, the spatial-temporal distribution of the grazing intensity of sheep can be obtained, which will provide basic data support for the study of forage–livestock balance and rotation grazing.

The traditional artificial observation method of sheep’s grazing behavior is time-consuming, laborious and subjective. Wang et al. [7] studied the grazing behavior of Euler-type Tibetan sheep in summer by tracking observation, and obtained a high correlation between grazing, rumination and rest time. With the development of GPS positioning technology, scholars began to use satellites to locate livestock, which facilitated the acquisition of the grazing behavior of cattle and sheep. Han et al. [8] obtained spatial-temporal distribution of grazing sheep under different stocking rates by attaching a GPS collar to the sheep combined with the remote sensing vegetation data at the typical degenerated grassland in Inner Mongolia. The relationship between walking distance and grassland conditions provides effective theoretical data in order to reduce grassland degradation and improve grassland ecology. Brennan et al. [9] and Rivero et al. [10] used GPS locators to distinguish grazing and non-grazing behaviors of cattle and analyze affected factors in grazing behaviors such as stocking rate, weather conditions, pasture topography and vegetation. It can also provide monitoring data of grassland utilization for managers and promote sustainable development of grassland. As the utilization of grassland by livestock is the main factor causing grassland ecological change, the unbalanced distribution of feed intake will lead to the decrease in the water content of grassland vegetation and the decrease of species number [11,12]. Therefore, effective assessment of it is of great significance to prevent grassland degradation and desertification. As for the acquisition method of feed intake, Laca et al. [13] recorded the sound signals of cattle grazing behaviors through a microphone tied to the forehead of cattle and identified the chewing and biting events to assess the feed intake of cattle. Zhang et al. [14] used the saturated alkane method to measure the feed intake of sheep, which provided the data basis for optimal grazing management.

In summary, due to the rapid development of the GPS positioning system, monitoring of grazing livestock movement trajectory by satellite positioning has been widely used, and accurate acquisition methods of grazing livestock feed intake have been developed. However, grazing trajectory data is not used to evaluate grazing intensity in a large area. In this study, a solar-powered GPS collar that can be worn by sheep was made, and the trajectory data of sheep was returned by GPRS (General Packet Radio Service) module of mobile communication. The correlation between different factors and the grazing intensity of sheep was explored based on the BP neural network model and the spatial-temporal distribution characteristics of the grazing intensity of grazing sheep were obtained.

## 2. Materials and Methods

**Figure 1:**
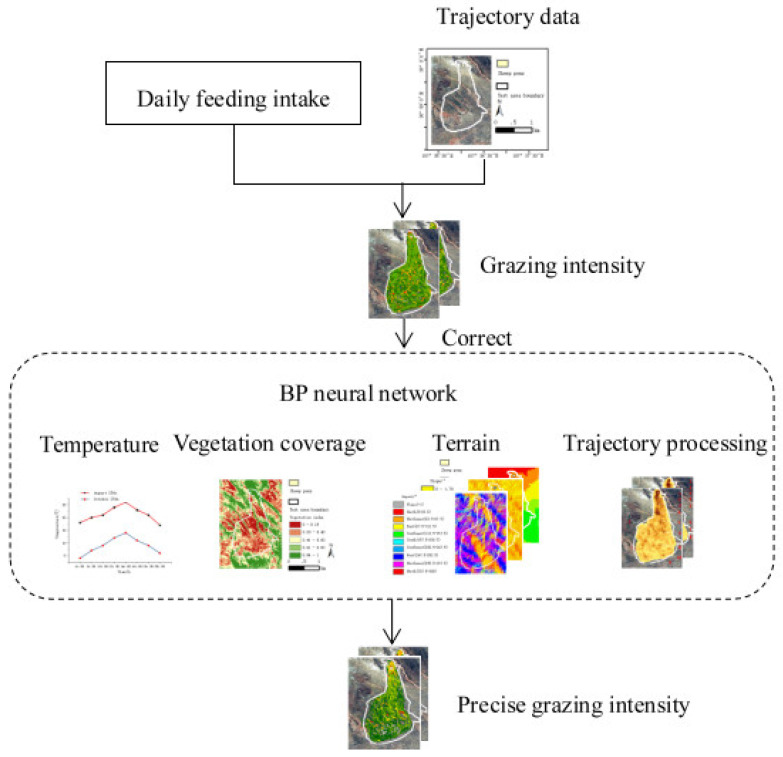
Overall flow chart of research scheme.

Figure 1 represents the technical route of the project. The daily feeding intake and trajectory data were used to calculate the spatial-temporal distribution of sheep grazing intensity; however, the accuracy of the grazing intensity calculated by this method was low. Therefore, the influence factors such as ambient temperature, topography of the test area, vegetation coverage and trajectory segments were considered to build a BP neural network fitting model, which achieved a high accuracy of spatial-temporal distribution of grazing intensity.

### 2.1. Overview of the Test Area

**Figure 2:**
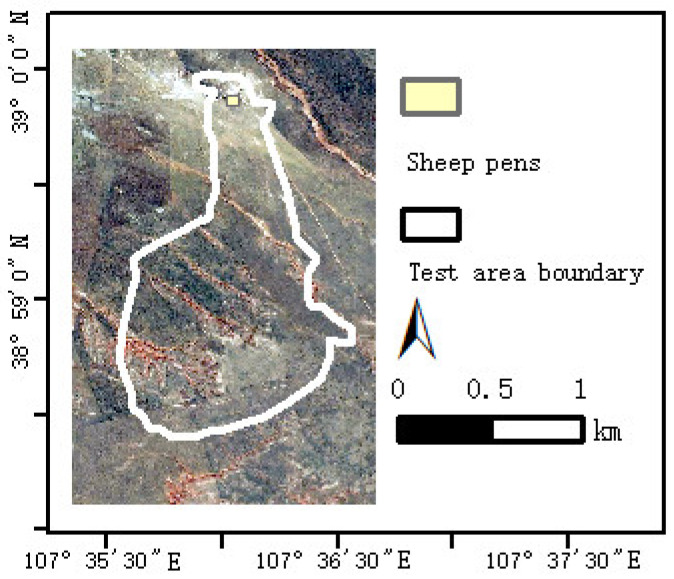
Location of the test area.

The test area is located in the desert grasslands of Wulanhua town to the west of Ordos city, Inner Mongolia. The grazing prohibition period starts from April to the end of June. In winter, the grassland is withered and mostly covered with snow, so August (summer) and October (autumn) are selected as the typical months for the data analysis of sheep grazing trajectory. The size of the test area is about 210 hectares; the climate is an arid and semi-arid continental climate zone with long and cold winters as well as short hot summers. The coordinate of the grazing grassland is from 38°58′22″ to 39°0′03″ of north latitude and from 107°35′20″ to 107°36′39″ of east longitude. The experimental area is shown in Figure 2, in which the sheep shed is located at the north corner of the grassland. There are about 200 sheep that leave the pen to graze at about 6 o’clock every morning and return to the pen from 6 to 8 o’clock in the evening. We selected two sheep from this flock as experimental subjects. GPS collars were installed on the two sheep.

### 2.2. Characteristics of Temperature in the Test Area

**Figure 3:**
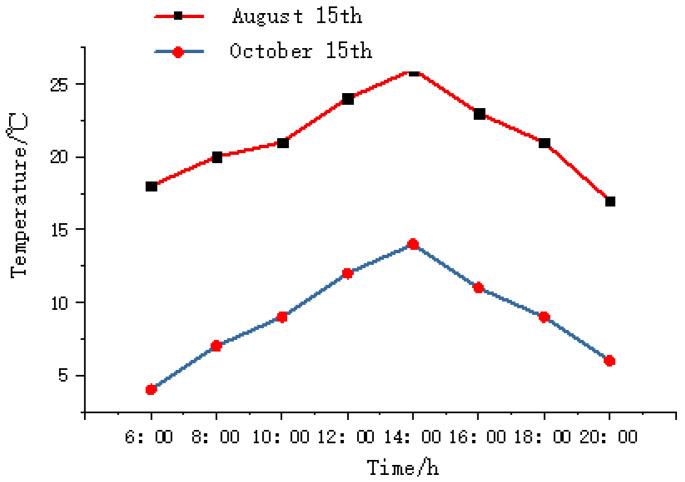
The temperature change curve of a certain day in summer and autumn in the experimental area.

Due to the poor heat resistance of sheep, the appetite of sheep decreases when the temperature is too high, hence the feed intake will decline or even stop. The daily temperature parameter is used as an influence factor of the sheep’s grazing behavior. Figure 3 is the temperature parameter change curves of the test area on August 15 and 15 October 2020, corresponding to summer and autumn, respectively.

### 2.3. Positioning Collar and Its Data Collection

**Figure 4:**
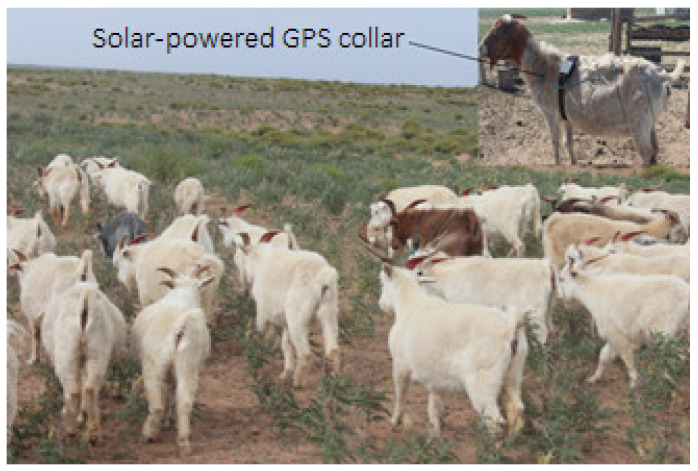
Test sheep with positioning collar.

As shown in Figure 4, the data acquisition terminal of this experiment was the positioning collar combining GPS and GPRS, which was powered by solar energy. The size of the terminal was 98 mm (length) × 64 mm (width) × 26 mm (height), and the weight was 167 g. The ratio of the positioning collar to the weight of sheep was less than 2.2%, so it would not affect sheep grazing [15]. The positioning data of the collar was collected by GPS, and the data was sent to the computer by a GPRS module, and the positioning accuracy is 2~10 m. The research results of Akasbi [16] show that the shorter the positioning interval is, the closer it is to the actual motion track. Therefore, the positioning interval of the collar was set to 2 min. Since it is powered by solar energy, the collar can work continuously. Summer and autumn are the main grazing seasons, the data of typical months August and October was collected for analysis, with a total of 17,358 records.

### 2.4. Vegetation Index in the Test Area

The test area is dominated by the subfamily of temperate desertification, and the main soil types are brown calcium soil and sandy soil. From the perspective of vegetation, the main group building species and dominant species are Artemisia giraldii pamp, Astragalus tataricus, Suaeda glauca and so on, and the main companion species is ice grass. Artemisia giraldii pamp has obvious main roots and many lateral roots, so it grows vigorously. It is the dominant plant species in the test area, has a diameter of 4~10 mm, and the flowering and fruit period is from July to October. On the hillside grassland and parts of sandy land, there are small fruits of Astragalus and Suaeda, which belong to the annual Chenopodiaceae plant that are rich in nutrients and loved by sheep.

**Figure 5:**
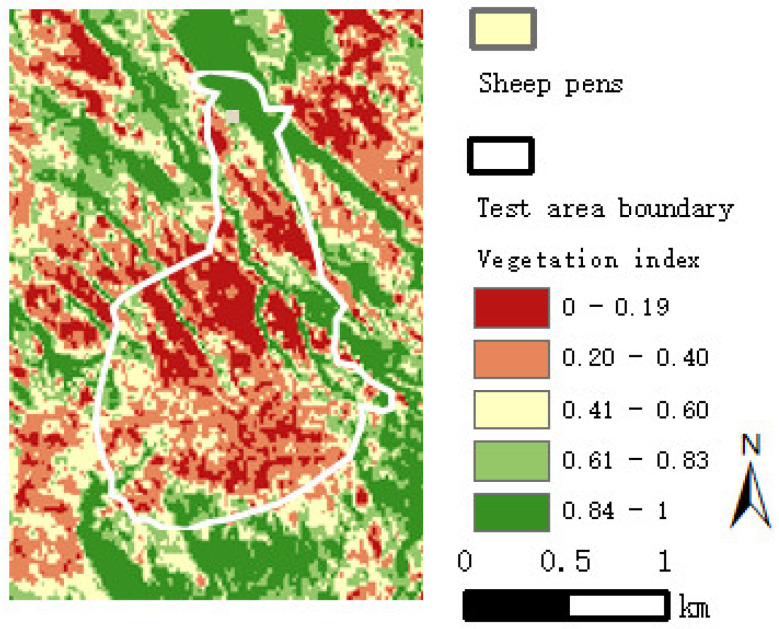
The NDVI map of the test area.

The remote sensing images of the Landsat 8 satellite are selected to extract vegetation parameters. The satellite has a total of 11 bands, with a spatial resolution of 30 m for bands 1~7 and 9~11, and a panchromatic band for band eight with a resolution of 15 m. Vegetation data analysis was carried out on the remote sensing image data on 16 October 2020. In order to ensure the accuracy of the data, radiometric correction and atmospheric correction were performed on the image data. The normalized vegetation index (NDVI) value of the test area was calculated by using the band operation method as follows:

(1) $$\mathrm{NDVI}=\frac{\rho_{NIR}-\rho_{R}}{\rho_{NIR}+\rho_{R}}$$

where $\rho_{NIR}$ is the spectral value of near infrared band, $\rho_{R}$ is the spectral value of red band. The NDVI value is between 0 and 1. The NDVI value is proportional to the vegetation coverage in the study area, and the NDVI value distribution in the test area is shown in Figure 5.

### 2.5. Topography Parameters in the Test Area

**Figure 6:**
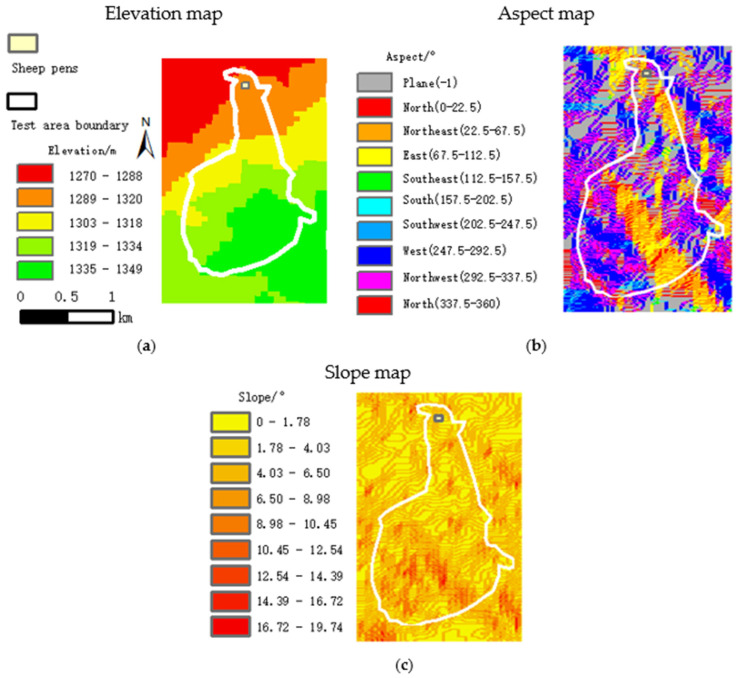
Topographic parameter maps of the test area. (**a**) Elevation map, (**b**) Aspect map, (**c**) Slope map.

Topography factors such as elevation, slope and other aspects in the test area have a certain impact on the grazing behavior of sheep [10]. The ASTER GDEM 30 m resolution remote sensing elevation image was selected to obtain the aspect, slope and elevation distribution of the test area as shown in Figure 6.

## 3. Preprocessing of Grazing Trajectory Data

### 3.1. Analysis of Aggregation Track Points Based on Kernel Density

**Figure 7:**
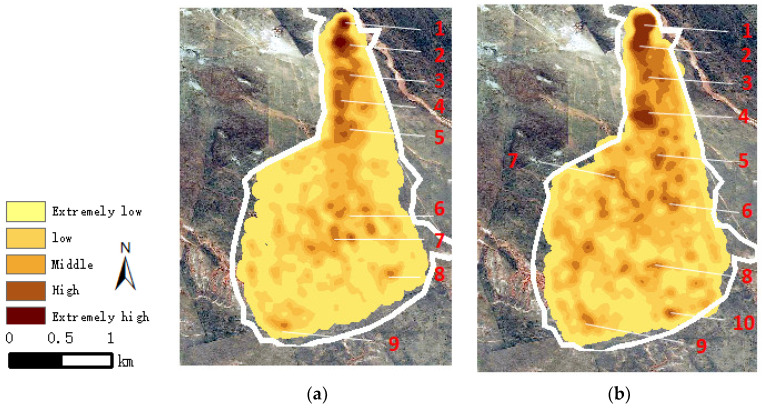
Kernel density map of sheep in summer and autumn. (**a**) Summer. (**b**) Autumn.

There are multiple aggregation track points for sheep in normal grazing locations, sheep pens and drinking water regions. The continuous trajectory of sheep in the rest area and drinking area has no influence on grazing intensity, so these two types of trajectory data need to be excluded. The kernel density analysis method is used to cluster the track points to obtain the rest area, drinking area and grazing area of sheep [17]. The track data points of the rest area and drinking area are eliminated to improve the accuracy of sheep grazing intensity assessment. The kernel density distribution map of tack points in summer and autumn is shown in Figure 7, where the trajectory data comes from two typical months, August and October.

The kernel density was divided into five grades, which were extremely low, low, medium, high and extremely high. The distribution of cattle represented by these grades decreased sequentially, and the darker the color was, the greater the distribution. Locations with density distribution of kernel density were numbered with numbers (1–9), where both 1 and 3 represented rest areas and sheep pens, 2 represented drinking water areas, and the remaining numbers represented areas where grazing behavior occurred. As shown in Figure 7, the numbers of positions 1, 2 and 3 on (a) and (b) were the same, which were the kernel density distribution of the rest and drinking area of sheep, and had no influence on grazing intensity. The vegetation index of the remaining gathering area (4~10) was at a high level. The vegetation index was from 58% to 90%, which was the main area for sheep grazing.

### 3.2. Buffer and Grid Configuration of Sheep Grazing Intensity Distribution

**Figure 8:**
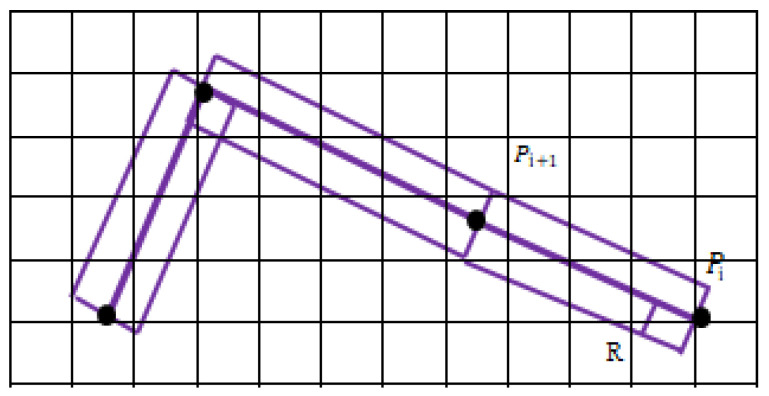
Schematic diagram of buffer and grid settings.

The grazing behavior of sheep is interactive, and there may be more than one sheep on the grassland in the same place. A buffer zone is used to describe the influence range or service range of the geospatial target. Therefore, a buffer zone can be established on the track line, which can be used to calculate the grazing area of sheep on the line segment of the trajectory point. Grid division is an effective way to measure the characteristics of sheep grazing behavior [18]. The test area is divided into small grids by grid division, and each small grid will carry the position, time information and grazing quantity information. The buffer and grid analysis diagram is shown in Figure 8. The grid size is set to a square grid of 13 m, and the buffer radius is a rectangular area of 3 m. The movement path of sheep is a sequence of time and position, where grazing trajectory *C* = [(*P*_1_, *T*_1_) (*P*_2_, *T*_2_) … (*P_i_*, *T_i_*) …] is a collection of spatial-temporal data.

## 4. Results

### 4.1. Spatial-Temporal Distribution Based on Constant Daily Feed Intake

Grazing intensity reflects the feeding status of sheep on the grass at a certain location. The grazing intensity can help grassland management departments to rationally and effectively utilize grassland and protect grassland ecology from damage. Generally, the sheep’s daily feed intake would not change significantly in one day, so the daily feed intake of sheep would not be affected by grazing intensity and vegetation index [8]. Assuming that the grazing speed of sheep is constant, the average daily grazing speed is equal to the average speed of a certain trajectory, and the average daily grazing speed of sheep can be calculated as follows:

(2) $$V_{ADFR}=\frac{I_{Z}}{T_{Z}}$$

where $V_{ADFR}$ (m/s) is the average grazing speed of a day, $I_{Z}$ (g) is the total grazing amount of a day and $T_{Z}$ (s) is the effective grazing time of a day.

Deqing et al. [19] showed that the daily dry matter intake of sheep grazing accounted for 2~4% of body weight. The total number of test sheep was 200, of which 70% were 40–60 kg, 15% were less than 40 kg, and 15% were more than 60 kg. Since the proportion of dry matter to fresh grass is about 39%, the daily intake of it is 7.6% of a sheep’s body weight. Therefore, in a day, 140 sheep ate 3.4~4.5 kg of grass, 30 sheep ate less than 3.4 kg of grass, and 30 sheep ate more than 4.5 kg of grass. The grid superposition method was used to superimpose the grazing intensity of the same grid with different trajectories, and the daily average grazing speed and daily effective grazing time were used to calculate the grazing intensity distribution of the trajectory segment within a day. The feed intake of the trajectory segment can be expressed as:

(3) $$I_{i}=V_{i}*T_{i}$$

where $I_{i}$ (g) is the grazing intake of the trajectory segment, $V_{i}$ (m/s) is the grazing speed and $T_{i}$ (s) is the elapsed time of the trajectory segment.

The grazing time of sheep can be divided into feeding time, rumination time, wandering time, standing time and other behavior time. The proportion (*a*) of feeding time to the total grazing time is from 84.92% to 90.19%, so the feeding time of a day is ($T_{Z}$ ∗ *a*) [20]. Choosing *a* = 86% and $V_{i}$ = $V_{ADFR}$, the feeding intake of the trajectory segment is:

(4) $$I_{i}=V_{ADFR}*T_{i}=\frac{I_{Z}}{T_{Z}}*T_{i}$$

So, the grazing intensity is:

(5) $$F=\frac{I_{i}\frac{C_{i}}{\sum \;C_{i}}}{S}=\frac{I_{Z}T_{i}C_{i}}{\sum \;C_{i}T_{Z}S}$$

where *F* (g/m^2^) is grazing intensity, $C_{i}$ is the number of trajectory points in the *i*-th cell, $\sum \;C_{i}$ is the total number of trajectory points in a day and *S* (m^2^) is the cell area.

**Figure 9:**
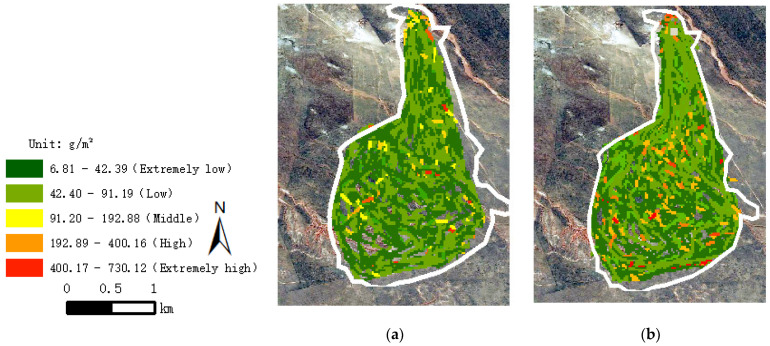
The grazing intensity distribution of sheep in typical months based on constant daily feed intake. (**a**) Summer. (**b**) Autumn.

Based on the above study on the algorithm of grazing intake distribution, using the trajectory data of August and October 2020 for analysis, the grazing intensity distribution of sheep was obtained as shown in Figure 9.

Figure 9 shows that the sheep depart from the sheep pens to graze around the pasture for a circle and then return. The grazing intensity on the path is not only relatively high but also accompanied by many mutations, and the superimposed effect of sheep’s grazing intensity on the path is not obvious in autumn. As a result, without considering the factors that affect the sheep grazing intensity, the distribution of sheep grazing intensity cannot be well represented only by estimating the daily feed intake and positioning data.

### 4.2. Spatial-Temporal Distribution of Grazing Intensity Based on Neural Network Model

As is known to all, sheep grazing intensity is affected by multiple factors, and the degree of influence of each factor is different. The factors that affect the grazing intensity are as follows: the duration time of the sheep’s trajectory segment (T), the walking distance (C), the slope of the test area (P), the elevation (E), the aspect (D), the vegetation index (NDVI), the sheep weight (S) and the temperature (F), which is a total of eight feature variables. So, a more accurate grazing intensity distribution model can be obtained by establishing a neural network model. We use the Neural Network Fitting Toolbox in MATLAB R2018b in our research. In order to ensure the unity of variable dimensions, the feature variables are standardized using the calculation formula as follows:

(6) $$X=\frac{X^{\#}-X^{*}}{\sigma }$$

where *X* is the standardized feature variable, $X^{\#}$ is the variable before preprocessing, $X^{*}$ is the mean value of the sample and σ is the standard deviation of the sample.

The BP (back propagation) neural network is a multi-layer feedforward neural network trained according to the error back propagation algorithm, and its calculation process is divided into a forward calculation process and a reverse calculation process. As the BP neural network is composed of an input layer, a hidden layer and an output layer, a total of eight variables affecting the grazing intensity are taken as the input layer and the grazing intensity variable as the output layer. A total of 780 trajectory points are selected as model samples, which are divided into a verification set (117), test set (117) and training set (546) according to the proportion of 15%, 15% and 70%. A correlation coefficient $R^{2}$ is used as the basis for judging the correlation between feature variables and the neural network model, and the calculation formula is as follows:

(7) $$R^{2}=1-\frac{\sum_{i=1}^{N}{\left(W_{i}-W_{i}^{1}\right)}^{2}}{\sum_{i=1}^{N}{\left(W_{i}-W_{i}^{2}\right)}^{2}}$$

where $R^{2}$ is the correlation coefficient, $W_{i}$ is the predicted grazing intensity value, $W_{i}^{1}$ is the actual grazing intensity value and $W_{i}^{2}$ is the average value of the predicted grazing intensity. It should be noted that the closer the correlation coefficient is to 1, the higher the degree of correlation. The mean square value is used to represent the error. The formula for calculating the mean square error between the actual value and the expected value is as follows:

(8) $$MSE=\frac{1}{N}\sum_{i=1}^{N}{\left(T_{i}-Y_{i}\right)}^{2}$$

where *MSE* is the mean square error of the expected value and actual value, $T_{i}$ is the expected value of grazing intensity and $Y_{i}$ is the actual output value of grazing intensity.

In order to facilitate future research and reduce the workload of follow-up research, we tried to narrow the range of influencing factors on the basis of ensuring accuracy. As shown in Table 1, nine combinations of multiple influencing factors were carried out to analyze the influence of each combination.

**Figure 10:**
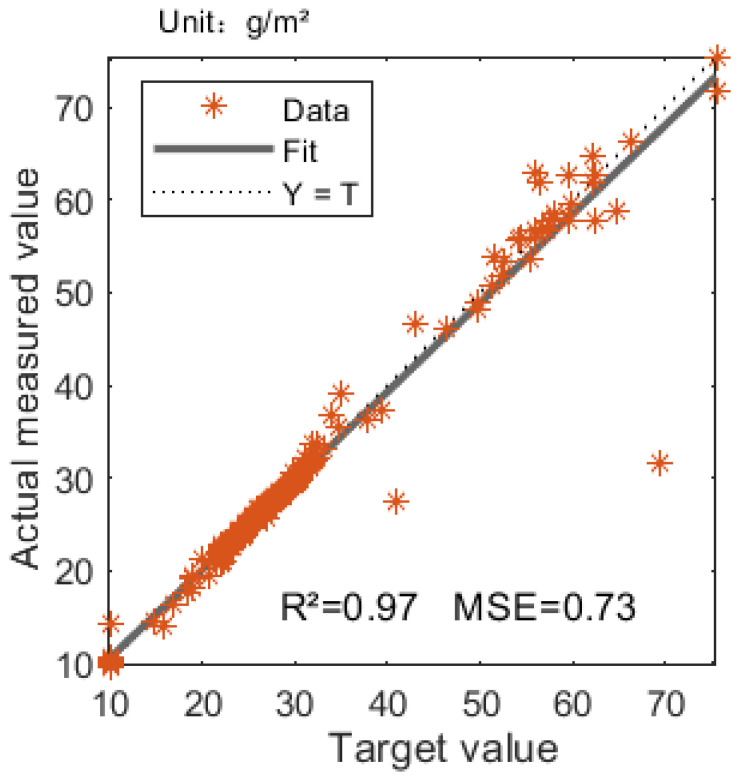
The evaluation of multi-factor combined BP neural network. The *X*-axis and *Y*-axis represent the target and actual measured values of grazing intensity. *Data* refers to the data set involved in the calculation, *Y = T* represents the ideal situation when there is no deviation between the measured value and the target value, and *Fit* represents the actual situation achieved by the model.

The grazing intensity neural model was trained and tested with different combinations of feature variables. When the input variables are vegetation index NDVI, sheep’s weight S, duration T, moving distance C and environment temperature F, the model is the best, and the correlation coefficient and mean square error of the model are $R^{2}$ = 0.97 and *MSE* = 0.73, as shown in Figure 10.

**Figure 11:**
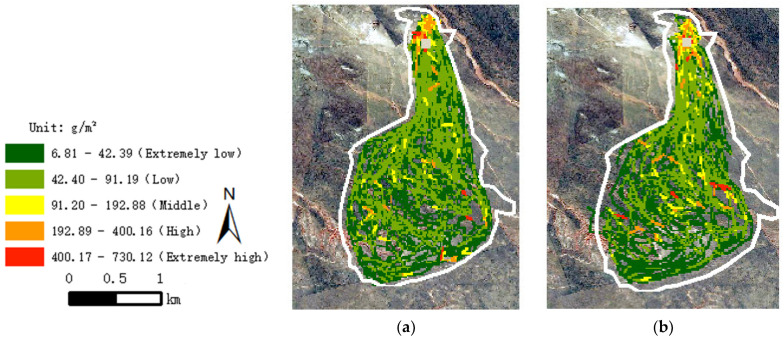
The grazing intensity distribution of sheep in typical months based on neural network model. (**a**) Summer. (**b**) Autumn.

According to the grazing intake obtained by the neural network model, the monthly grazing intensity of sheep in summer and autumn was analyzed. The monthly grazing intensity of sheep in summer and autumn is shown in Figure 11.

As shown in Figure 11, the grazing intensity of sheep in summer was higher than that in autumn, which was obvious on the south of the test area. Moreover, using the neural network, the distribution of grazing intensity showed a uniform transition, and the repeated grazing area of sheep was superposed, which could better reflect the situation of grazing intensity in different locations. The monthly grazing intensity in the test area was 6.81~730.12 g/m^2^, without considering irrelevant data near the sheep pens and drinking water region.

## 5. Discussion

In view of the unreasonable use of grasslands such as overgrazing in Inner Mongolia, with the more and more comprehensive application of computer and GPS positioning technology in animal husbandry and grassland science, GPS technology is used to determine the spatial-temporal distribution of grazing intensity. On the basis of obtaining trajectory data, the study on the spatial-temporal distribution of the grazing intensity of sheep can provide a relevant theoretical basis for grassland supervision departments to formulate management systems.

The purpose of this study was to explore the true grazing intensity of sheep in a specific area and evaluate grazing intensity using grazing sheep trajectory data. However, experiments and studies have found that the grazing behavior of sheep in the real environment is affected by many factors, such as the duration of the sheep’s trajectory segment (T), the walking distance (C), the slope of the test area (P), the elevation (E), the aspect (D), the vegetation index (NDVI), the sheep weight (S) and the temperature (F). In order to obtain an accurate fitting model of the grazing intensity of sheep, the BP neural network is used to analyze these factors. The result shows that there are five factors that have a greater impact, namely vegetation index, sheep weight, duration, moving distance and ambient temperature, and the coefficient of determination of the new model is $R^{2}$ = 0.97, and the mean square error is *MSE* = 0.73. It is proved that the correlation is strong and the error is small. After that, the grazing intensity data is registered to form a picture with strong observation and readability.

With the BP neural network as the core, this project explores the main factors and combinations of main factors that affect the grazing intensity of sheep, and it analyzes the grazing intensity of sheep. In the follow-up, we will continue to study the grazing behavior of sheep to better measure the behavior of sheep.

## 6. Conclusions

There are many factors that affect the grazing intensity of sheep. The factors in this study are the duration of sheep movement track segment, walking distance within the duration, slope, elevation, slope aspect, vegetation index, sheep weight and environment temperature. According to the model analysis, the factors that have a greater impact on the grazing intensity of sheep were identified as: vegetation index, sheep weight, duration, moving distance and ambient temperature. According to these five factors, a BP neural network model was established to fit the feeding intensity. The coefficient of determination of the new model was 0.97, and the mean square error was 0.73. These two parameters indicate a high degree of accuracy of the model. Therefore, the monthly grazing distribution of sheep was established through this model, and the result showed that the grazing intensity in summer was greater than that in autumn; the feeding intensity of sheep was concentrated between 6.81 and 730.12 g/m^2^.

## Figures and Tables

**Table 1:** Collocation of factors.

Serial Number	Vegetation Index	Sheep Weight	Duration	Temperature	Moving Distance	Slope	Aspect	Elevation	$R^{2}$	*MSE*
X1	1	1	1	1	1	1	1	1	0.95	31.03
X2	1	1	1	1	0	0	0	0	0.96	1.51
X3	0	0	0	0	1	1	1	1	0.92	2.2
X4	1	1	0	0	1	1	1	1	0.9	0.000058
X5	1	0	1	0	1	1	1	1	0.95	0.018
X6	0	1	1	1	0	1	1	1	0.9	0.03
X7	1	1	1	1	1	0	0	0	0.97	0.0048
X8	1	1	1	1	1	1	0	0	0.91	0.0049
X9	1	1	1	1	1	1	1	0	0.95	28.12

## Data Availability

Not applicable.
